# Evaluating and refining undersea cable path planning algorithms: A comparative study

**DOI:** 10.1371/journal.pone.0315074

**Published:** 2024-12-26

**Authors:** Tianjiao Wang, Zengfu Wang, Bill Moran, Xinyu Wang, Moshe Zukerman

**Affiliations:** 1 Center for Intelligent Multidimensional Data Analysis Limited, Hong Kong Science Park, Hong Kong SAR, China; 2 School of Automation, Northwestern Polytechnical University, Xi’an, China; 3 Department of Electrical and Electronic Engineering, The University of Melbourne, Melbourne, VIC, Australia; 4 Department of Electrical Engineering, City University of Hong Kong, Hong Kong SAR, China; King Fahd University of Petroleum & Minerals, SAUDI ARABIA

## Abstract

This paper compares three automated path-planning algorithms based on publicly available data. The algorithms include a Dijkstra-based algorithm (DBA) that improves on the straightforward application of Dijkstra’s algorithm, which restricts the path only to the grid edges. We present a fair and comprehensive comparison method for evaluating multiple algorithms—DBA, the Fast Marching Method (FMM), and a great circle-based method. To evaluate the performance of automated path-planning methods, we compare them based on two main criteria: (1) the total weighted cost which is a combined measure of various costs and risks of the cable path according to their weights, and (2) the algorithm’s runtime. FMM achieves a proven minimal weighted cost cable path solution given the data. This is not the case for the other two alternatives. On the other hand, DBA may have a runtime advantage over FMM. The paper discusses the sensitivity of DBA and FMM to diagonal configurations and to variation in the triangulation of the manifold, finding that DBA is more significantly affected by these factors than FMM. Furthermore, we explore how cable direction metrics can influence the performance of these methods. Through this comparative analysis, we aim to provide insights into the efficiency and effectiveness of these methods in practical scenarios and provide a useful reference for the industry in choosing the best approach for automatic cable path planning software.

## 1 Introduction

Undersea cable path planning is a complex process that aims to determine the optimal path between two or more specified locations based on various design considerations. These considerations typically include factors such as latency constraints, transmission capacity, avoiding disaster-prone areas, and minimizing interference with fishing areas [1–3]. The objective is to find a path that minimizes a cost function which is a summary of various cost functions associated with the relevant design considerations. The great circle-based method is a commonly used reference method in industrial software applications. For example, the industry-leading submarine cable path planning software, Makiplan, employs the great circle-based method to obtain an initial path. Cable designers then use this path to send ships to survey the seabed environment, thereby determining the cable-laying path. The Dijkstra’s algorithm and the Fast Marching Method (FMM) have both been widely considered in research for cable path planning [1–5]. Both methods are capable of providing optimal paths. The Dijkstra’s algorithm finds the optimal path based on the data by operating within the constraints of grid edges in the graph, while FMM determines the optimal path using the given data without being restricted to grid edges. Automated cable path planning based on publicly available data provides surveyors with an initial path along which more data can be collected.

### 1.1 Background of submarine cable path planning

Our focus is on submarine cable path planning, with the goal of identifying an optimal route where costs and risks are minimized. The cost of a cable depends on the length, ground/soil condition, the requirement for security arrangements, licensing, and various other factors [6].

Deep ocean waters where the depth exceeds 200 meters are the largest habitat for life on Earth and the most difficult to access [7]. In deep waters, comprising over 90% of the entire ocean area, environmental information is usually obtained at a kilometer-scale resolution [8]. In shallower waters, other methods can achieve up to a meter- and even centimeter-scale resolutions [8]. Publicly available data is generally of lower resolution than data collected by surveyors, especially in shallow waters [9]. The cable path generated by the automated path planning method serves as an initial guideline or benchmark rather than the final route for cable designers. This software provides surveyors and designers with a preliminary path, allowing them to restrict their data collection to regions in the vicinity of the chosen path(s). As we demonstrate in this paper, the results of FMM and DBA provide lower-cost cable path solutions than the result of the great circle approach. Accordingly, they can be used to provide an initial path instead of the latter, which is currently used in practice for this purpose.

### 1.2 Path planning methods

Current industry practice is to perform cable routing manually for much of the design process, though MakaiPlan software is widely used to assist in this process [10]. MakaiPlan does not provide automated optimization for path planning, rather it finds the shortest distance along the great circle “as a series of Rhumb lines” [10]. This path is then modified manually to reduce costs associated with risk and other factors. The length of long-haul cables may be thousands of kilometers, so manual path planning in the absence of a scalable, automated software tool is costly and, importantly, may significantly fail to achieve the desired optimal cost-risk trade-off. Automated submarine cable path-planning methods should not only assess issues of the cost of cable laying, but also consider avoidance areas, and risks by using related penalties in the objective function. The advantage of the automated submarine cable path-planning methods is they provide a good initial path to facilitate more frugal and focused data collection. The private data thereby collected can also be used automatically to generate and improve the cable path, which can then be used by the designers as a benchmark, ultimately resulting in an improved quality of the cable path at a lower cost to the path design process. Then, the data collection in the vicinity of the initial high-quality path allows automated cable path planning to produce further improvements that complement and assist the manual approach. Analyzing and comparing automatic cable path planning methods can provide a useful reference for the industry in choosing the best approach for automatic cable path planning software.

Apart from the relatively simple great circle-based method, the two other algorithms that have been widely discussed in the context of cable path planning are the FMM and Dijkstra’s algorithm. Given the importance of developing automatic path-planning tools, it is, therefore, important to enhance our understanding of the performance of these algorithms and how they compare with each other.

Using Dijkstra’s algorithm for cable path planning over a triangulated irregular network with a given set of grid-point (node) values is based on updating the value of a node by summing the value of an adjacent node and the value of the edge connecting the current node and the adjacent node. While FMM numerically solves the partial differential Eikonal equation to update the value of a node. FMM consumes more time than DBA, although both algorithms have polynomial complexity [11–13]. A limitation of Dijkstra’s algorithm is that it restricts the geometry of links that the path can take to a sequence of edges between network vertices, though manual adjustment would, inevitably, smooth the path from Dijkstra’s algorithm. Unlike the “quantization” necessary for the implementation of Dijkstra’s algorithm that forces the path to walk exclusively along the edges of the network, FMM can find a lower path cost result by considering the costs in all points of interest on the manifold, allowing the path to traverse through the interiors of the grid. FMM can be regarded as a continuous version of Dijkstra’s algorithm and the resulting path *together with its length* is proven to converge to the continuous physical solution as the grid resolution tends to zero [11, 12]. In practice, the limit is not achieved since the distance between adjacent grid points is significant, so the step size can never approach zero, even for FMM. Recall that at any stage, the resolution is based on the available data. As previously mentioned, automated cable path planning methods offer surveyors an initial route to focus the acquisition of additional data. However, both FMM and the Dijkstra’s algorithm have limitations. Their reliance on publicly available data can constrain the quality of the resulting cable paths. Another notable limitation is that neither algorithm accounts for the cable’s curvature [14] requirement to avoid sharp corners, ensuring the cable’s integrity and functionality.

### 1.3 Motivation and contributions of the study

The following describes the motivation and contribution of this paper.

- Firstly, we describe an application of the Dijkstra’s algorithm, called here *the Dijkstra-based algorithm* (DBA), that overcomes the limitation of the path results from a straightforward application of the Dijkstra’s algorithm to the graph consisting of only the grid edges. The DBA accomplishes this by adding diagonals of various lengths.
- Moreover, this paper introduces, for the first time, a fair and comprehensive approach for evaluating and comparing the algorithms DBA, FMM, and the great circle-based method. This comparison approach takes into account two key issues: the quality of the cable path (cable weighted cost), and the runtime complexity, to ensure fairness and comprehensiveness in the comparison. We use a Fibonacci heap min-priority queue to achieve DBA with a runtime complexity of Θ(|*E*| + |*N*| log |*N*|) [13], where |*E*| is the number of edges and |*N*| in the number of nodes of the graph. We calculate the runtimes of the FMM and DBA on maps of different resolutions and compare the quality of their cable path results. Beyond the common approach that compares algorithms in terms of their performance and their running times [6, 11], the uniqueness of our approach is that we address the problem of how to compare fairly between the various algorithms in areas where there is a lack of high-resolution measurement data. In a low-resolution map where information is limited, relying only on the resulting cost as a measure of performance may lead to significant risk areas along the cable path being ignored because of a lack of data, even if the objective function includes risk penalties. To address this problem, we use the same path obtained based on the low-resolution map in a high-resolution map, enabling a re-evaluation of the cost using the high-resolution data. By doing so, we can conduct a more comprehensive assessment of the cable path, taking into account finer details and reducing the likelihood of ignoring critical risk areas.
- Furthermore, we achieve improvement in the results for DBA by adding diagonal edges to the grid as appropriate, based on very rough information about the direction the cable will follow. While FMM is optimal given the data, DBA is limited to edges between the grid points. If the edges are selected so that they coincide with the optimal path obtained by FMM in the manifold, DBA will perform well, though, of course, this presupposes some knowledge of the optimal path we are trying to find.
- The numerical results demonstrate that FMM achieves the best path-planning solution with minimum weighted cost of all three methods while DBA has lower runtimes than FMM. Notice that, as the resolution is limited, especially in deep waters, the difference in the runtimes may not be a significant concern, as parallel processing solutions for cable path planning are available for both FMM [15] and the Dijkstra’s algorithm [16]. These results are consistent with the theoretical results in [11, 12]. We also demonstrate that the path obtained by the great circle-based approach has the lowest quality of the three. This is expected because it ignores the Earth’s surface and all potential obstacles and risky areas along the path. As we have mentioned in the section above, the comparison results provided in this paper can provide important guidance on choosing between these algorithms in developing automated cable path-planning software.

The remainder of this paper is organized as follows. In Section 2, we describe current automated methods for submarine cable path planning. Next, in Section 3, we explain the model of the Earth’s surface we use for applying these automated methods and introduce our comparison strategy for different path-planning approaches. We provide numerical results on the performance of the three algorithms considering various data density resolutions in Section 4. Then, in Section 5, we discuss and summarize the characteristics of the FMM and DBA algorithms, along with the scenarios where they are most suitable. Finally, we conclude the article in Section 6.

## 2 Related work

Here we discuss existing research on automated methods for submarine cable path planning that optimize objectives associated with cost and risk and meet the quality of service (QoS) requirements. Among the related literature, FMM, Dijkstra’s algorithm, and heuristic searching methods have been used in cable path planning.

Zhao *et al*. [17] provided a raster-based path analysis that minimizes the accumulative path cost. They considered both cable lengths and earthquake risks, with a model combining a 3D landform map and an earthquake likelihood map. They used Dijkstra’s algorithm for path planning with a cost function that included costs associated with cable length and earthquake risk.

Compared with the classical Dijkstra’s algorithm that finds the shortest path between nodes, typically in 2D, [16] proposed a method involving constraints on the cable path planning process to avoid undesirable regions. They pointed out that the bending stiffness of the submarine cable or pipeline and the maneuverability of the plows are non-negligible. In addition, the numerical solver based on their method was implemented on parallel architectures, which can provide optimal paths of real-life examples in acceptable computational times. However, their approach is still based on Dijkstra’s algorithm, where the path-planning solution is restricted to either lateral or diagonal links between grid nodes. They did not discuss how adding the diagonals might influence the performance of the Dijkstra algorithm. Their focus was on controlling the curvature of the cable. The limitations of Dijkstra’s algorithm, as present in both [16, 17], are not addressed in their work but have been overcome and improved upon in this research.

Huang *et al*. [18] presented a method for designing submarine cables, where restrictions on the motion of an autonomous underwater vehicle were incorporated in the constraints, and an A* algorithm was used to optimize. They utilized a 2D grid map to represent the surface of the Earth. Similar to Dijkstra’s algorithm cable path planning approach, the designed cable path of their method was restricted to the edges of the grid.

Wang *et al*. [2] considered the cable path planning problem using a triangulated irregular network to model the Earth’s surface, and running the Dijkstra’s algorithm with the interval-partition-based label-setting approach. In [19], FMM is applied for cable path planning in real-world size problems where varying levels of cable shielding are part of the risk reduction strategy to enhance earlier work on this topic in [2].

Wang *et al*. [1] presented an FMM-based approach to solve the multi-objective cable path planning problem on the Earth’s surface. As mentioned before, FMM is a path-planning approach that avoids the weaknesses of Dijkstra’s algorithm [11, 12]. This approach includes two phases: the first phase starts from a source node *N*_*s*_ and considers other nodes in order according to their cost from the source node. This phase stops when the “wavefront” [11, 12] which represents the set of points with equal cost reaches the destination node *N*_*d*_. The second phase tracks back from *N*_*d*_ to the source node *N*_*s*_ using the cost information of each grid node calculated in the first phase. The final path backward is obtained by tracking backward from *N*_*d*_ to *N*_*s*_ along the steepest gradient.

Wang *et al*. [20] used Simulated Annealing (SA) to optimize weights of the design considerations of [1] to minimize the Fréchet distance between existing cable paths and paths with minimized total life-cycle cost obtained by FMM. A new approach, named FMM/SA, has been proposed, and its usefulness is demonstrated as an automatic cable path planning approach by comparing the path it generates to a real-life long-haul cable path between the same two end nodes. In the studies discussed above [1, 2, 20], the focus was primarily on the performance of FMM in cable path planning, without considering the runtime or comparing its performance with other algorithms.

In a recently published paper [21], Zhao *et al*. associated the pheromone and heuristic functions of the ant colony optimization algorithm to the geographical map of costs and risks, enabling it to explore a submarine cable route through multi-objective optimization. However, such methods, being heuristic, do not have the rigorous justification for finding the optimal path that methods such as FMM and Dijkstra’s algorithm do. While we have not done simulations for the ant colony optimization algorithm, we suspect that they are also slower than FMM and Dijkstra’s algorithm.

Wang *et al*. [6, 22] also addressed the cable path planning problem as a multi-objective optimization problem including, as additional costs, the cable breaking risk and the laying cost, also taking into account various design considerations and multiple design levels for cable shielding. Then, the problem was converted to a single objective optimization problem by using a weighted sum (Pareto) method. [22] introduced volcano eruptions, steep slopes, and marine protected areas constraints to FMM while designing cost-effective and reliable cable paths by using an infinite value for the risk objective of the problematic areas. In [23], more up-to-date information on FMM-based cable path planning and a video that demonstrates a tool based on FMM for cable path planning are provided. In [6], FMM and Dijkstra’s algorithm were used for long-haul cable path design, showing that the FMM-based method offers superior path planning solutions compared to Dijkstra’s algorithm. In particular, a cost-benefit of up to 17.5% for FMM over Dijkstra’s algorithm in cable path planning in a realistic example was demonstrated in [6]. These results were based on an implementation of Dijkstra’s algorithm where no diagonals were added to the grid cells with four sides, each connecting adjacent grid nodes in the *gridded graph*.

In this paper, similar to the research in [6, 22], a *gridded graph* is based on a latitude/longitude grid of equally spaced points, where the nodes are the grid points on the Earth’s surface for which data is available. The basic edges connecting adjacent points are segments of lines of latitude and longitude. Beyond the previous research [6, 22], additional diagonal edges also be adjoined, as we will describe below. Note that lengths of edges need not be Euclidean lengths, as they may include factors such as risk.

To the best of our knowledge, no existing publication provides a comprehensive comparative analysis of these approaches that considers the advantages of Dijkstra’s algorithm in utilizing grid cell diagonals. Additionally, there is no in-depth comparison of the performance of FMM and DBA in terms of resolution, sensitivity to diagonal configurations and variations in manifold triangulation, or sensitivity to cable direction.

## 3 Problem modeling and description

Here we describe the utilization of triangulated manifolds and gridded graphs to represent the Earth’s surface and outline the key aspects in which we will compare FMM and DBA.

### 3.1 Problem modeling

**Fig 1:**
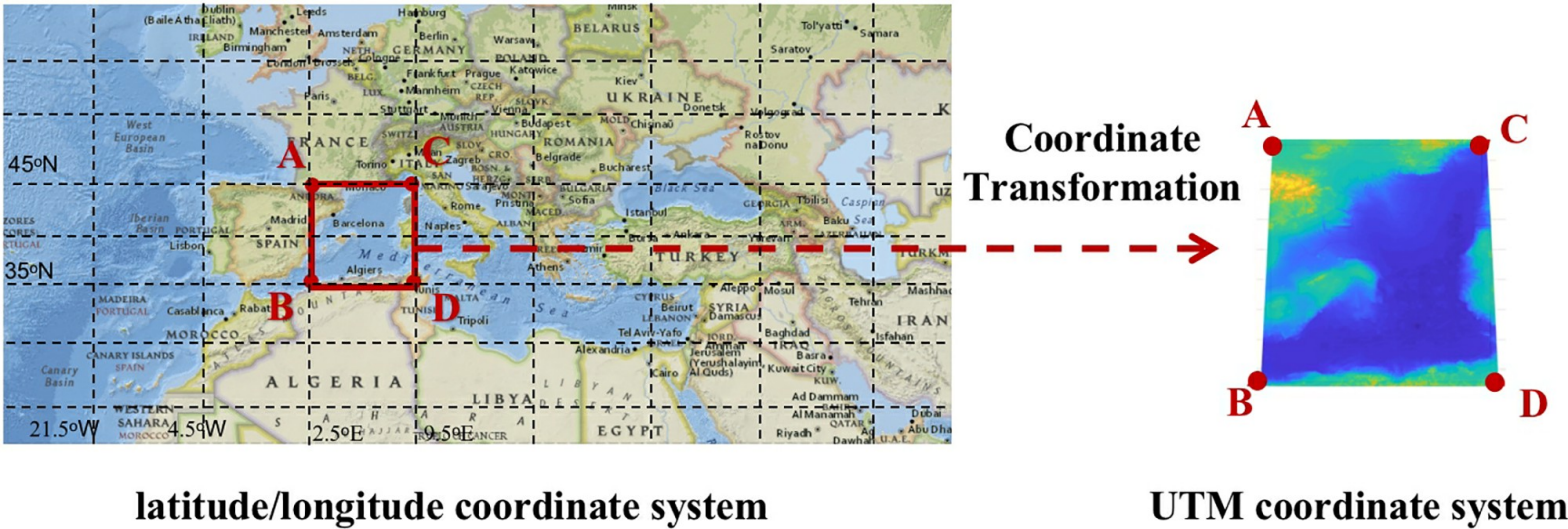
Coordinate system transformation. Source: USGS National Map Viewer.

We start with a grid of data points. In the area of interest, which is our selected area to search for the optimal path between the two end nodes, we consider the grid points that are equally spaced along the longitude and latitude. These are the grid points points at which data has been collected. In this paper, we use the term *grid cell* to refer to a four-edge shaped cell, each connecting adjacent grid points. The data allows us to assess issues like the cost of cable laying, avoidance areas, and risk, as discussed above, and to apply a weighted cost to each node, which is referred to [22]. In addition, a coordinate transformation is applied to our area of interest. We convert each grid point from latitude/longitude coordinates to Universal Transverse Mercator (UTM) coordinates. The weighted cost in each node remains the same. As shown in Fig 1, nodes A and C have the same latitude while their longitude difference is 8.5°, similarly for nodes B and D. The four are the vertices of a grid cell. However, in the UTM coordinates, the distance between nodes A and C is shorter than between nodes B and D. Accordingly, the grid cells are not always of a rectangular shape. The distortion in this projection is reduced close to the equator where the cells are closer to being rectangular shaped, or even a square shared if the distance between adjacent points of the longitude and the latitude directions are the same.

We refer to the data points as *data nodes* or, when no confusion arises, just nodes, and we write RG for the set of these nodes in the region of interest. For a node *N*_*i*_(*x*_*i*_, *y*_*i*_) ∈ RG, its weighted cost is *c*(*N*_*i*_), representing the various costs/risks per unit length of cable that need to be assigned to cables laid in the vicinity of the node *N*_*i*_ following the consideration in [1]. Other issues, such as volcanoes, fishing areas, license requirements, and the slope of the surface, can be added if such information is available. The total weighted cost used in the optimization of the cable *γ* is C(*γ*), which is the objective function cost that the algorithms aim to minimize. It also serves as one of the performance metrics used for comparing these algorithms. We write

$$\begin{array}{c} \text{C}\left(\gamma \right)=\int_{0}^{l(\gamma )}c\left(\gamma \left(s\right)\right)ds. \end{array}$$ (1)

For DBA, nodes on the gridded graph are connected by edges in three possible ways, resulting in what we call Gridded Graph I (GG-I), Gridded Graph II (GG-II), and Gridded Graph III (GG-III). For a basic gridded graph, edges connect the adjacent nodes of the grid cell aligned with latitude and longitude, respectively. Thus (*x*_*i*_, *y*_*i*_) is connected to (*x*_*i*±1_, *y*_*i*_) and (*x*_*i*_, *y*_*i*±1_).

**Fig 2:**
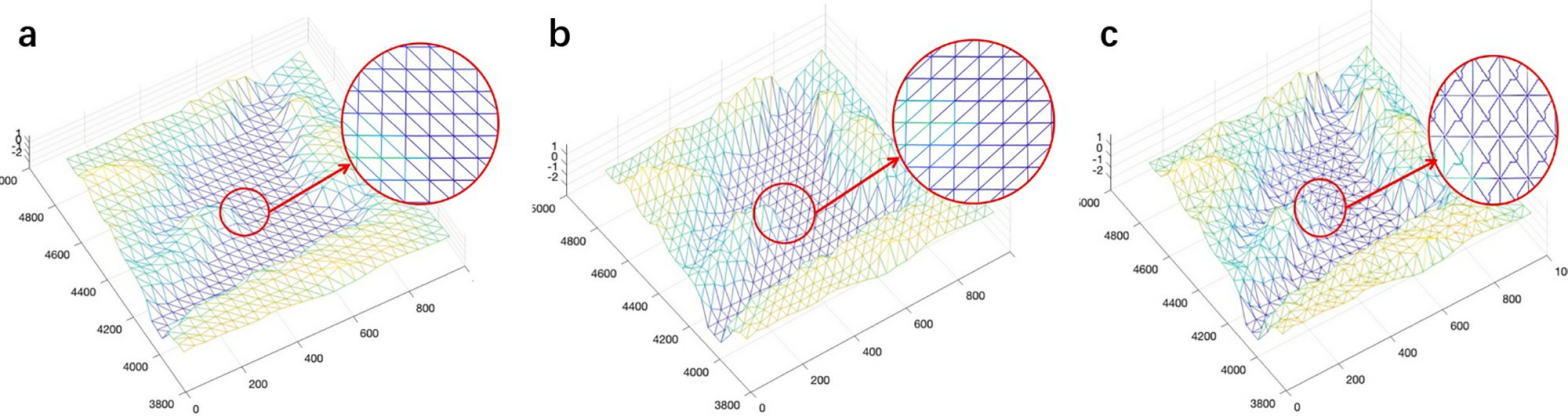
Different manifolds and gridded graphs. (a) TM-I & GG-I. (b) TM-II & GG-II. (c) GG-III.

In the case of GG-I, we add to the edges of the basic gridded graph a diagonal edge from (*x*_*i*_, *y*_*i*_) to (*x*_*i*+1_, *y*_*i*+1_) in each case, except at the borders of RG, where one of the nodes does not exist, as appropriate. While in the case of GG-II, we add to the edges of the basic gridded graph a diagonal edge from (*x*_*i*+1_, *y*_*i*_) to (*x*_*i*_, *y*_*i*+1_) in each case. For GG-III, we add, in addition to the edges added in GG-II, the diagonal edges (*x*_*i*_, *y*_*i*_) to (*x*_*i*+1_, *y*_*i*+1_). GG-I, GG-II, and GG-III are shown in Fig 2.

We defined the cable path from DBA as *γ*_*D*_; *γ*_*D*_ is the sum over the edges that form the path; C(*γ*_*D*_) represents the total cost of the cable path, defined by the following function.

$$\begin{array}{c} \text{C}\left(\gamma_{D}\right)=\sum_{i}c\left(e_{i,j}\right)*\alpha_{i,j}, \end{array}$$ (2)

where *e*_*i*,*j*_ is the edge between nodes *N*_*i*_ and *N*_*j*_, *α*_*i*,*j*_ = 1, means edge *e*_*i*,*j*_ is selected by the DBA and in the path *γ*_*D*_, otherwise, *e*_*i*,*j*_ is not in the path *γ*_*D*_. *c*(*e*_*i*,*j*_) is the weighted cost of the edge *e*_*i*,*j*_ as defined in Eq (3). In addition, *c*(*e*_*i*,*j*_) is symmetrical, i.e., *c*(*e*_*i*,*j*_) = *c*(*e*_*j*,*i*_), which can be directly used for DBA.

$$\begin{array}{c} \begin{array}{cc} c(e_{i,j}) & =\sqrt{{\left(x_{i}-x_{j}\right)}^{2}+{\left(y_{i}-y_{j}\right)}^{2}+{\left(z_{i}-z_{j}\right)}^{2}}\\ & \times \frac{c\left(N_{i}\right)+c\left(N_{j}\right)}{2}, \end{array} \end{array}$$ (3)

where (*x*_*i*_, *y*_*i*_) and (*x*_*j*_, *y*_*j*_) are the coordinate of nodes *N*_*i*_ and *N*_*j*_, *z*_*i*_ and *z*_*j*_ are the altitudes of nodes *N*_*i*_ and *N*_*j*_, and *c*(*N*_*i*_) and *c*(*N*_*j*_) are the weighted cost of nodes *N*_*i*_ and *N*_*j*_, respectively.

We implement the FMM by modeling the region of interest by a triangulated manifold [11, 12] with nodes of RG, so that each grid cell is divided into two triangles. There are two alternatives for each grid cell, and we make this choice arbitrarily. We consider two different triangulated manifolds in this paper as shown in Fig 2a and 2b, named Triangulated Manifold I (TM-I) and Triangulated Manifold II (TM-II). For FMM, applied directly to the triangulated manifold, we defined the resulted cable path as *γ*_*F*_, and we write C(*γ*_*F*_) as

$$\begin{array}{c} \text{C}\left(\gamma_{F}\right)=\int_{A}^{B}c\left(\gamma_{F}\left(s\right)\right)ds. \end{array}$$ (4)

The cable *γ*_*F*_ obtained by FMM is naturally parametrized by arc length *s*, and *c*(*γ*_*F*_(*s*)) can be obtained by using the linear interpolated cost for the triangular regions between grid nodes; that is, the cost at a point inside the triangular region is defined to be the convex combination of the costs at the grid nodes that form the vertices of the triangle, where the coefficients are determined by the relative distances to those grid nodes. Table 1 provides descriptions for notations used in this paper.

**Table 1:** 

Table of Notation	
TM	Triangulated manifold in $R^{3}$
GG	Gridded graph in $R^{3}$
*N*_*i*_	Data nodes in the discretized region
RG	The set of data nodes in the region of interest
(*x*_*i*_, *y*_*i*_)	Coordinates of node *N*_*i*_
*c*(*N*_*i*_)	Weighted cost at the location of node *N*_*i*_
*e*_*i*,*j*_	Edge connecting two nodes *N*_*i*_ and *N*_*j*_
*γ*	Geodesics of the cable path
*c*(*e*_*i*,*j*_)	Cable cost of the edge *e*(*i*,*j*)
*C*(*γ*)	Cost of the cable

### 3.2 Problem description

We first compare the performance of FMM and DBA on various resolution maps. Increasing map resolution improves the accuracy and quality of the resulting cable path and system design because the additional information in the refined map may point to additional risks that are invisible in the lower-resolution map. Accordingly, the cable planning method applied in higher resolution maps may improve the quality of results relative to the lower resolution map.

The cable routing industry is using MakaiPlan [10] widely to provide an initial path for sending a boat to collect more data based on great circles, so it is important to make a quantitative comparison of this with the published cable path planning solutions using FMM and DBA. As the resolution may affect the performance of automatic path planning, it is important to assess the effect of resolution on the comparison between the three approaches: FMM, DBA, and the great circle-based method.

In addition to resolutions, another factor that may mainly affect the performance of DBA is related to the difference between the direction of the intended path, assuming a 2D presentation of the Earth, and the direction of the side (for each triangle in the gridded graph) whose direction is closest to that of the direction of the intended path. As an extreme example, consider the case when we have the two endpoints positioned in the southeast and northwest corners, respectively. If we use DBA to connect the grid points of the gridded graph, and all the diagonals in each grid cell that are directed southeast-northwest are sides of triangles of the gridded graph, this will improve the performance of DBA over a gridded graph in which only the northeast-southwest diagonals are included. This is a relatively crude adjustment and will not achieve an improvement in many directions. In addition to adding diagonals within a single grid cell, we can also add diagonals across two or even multiple adjacent grid cells. In this way, the angle of the diagonals relative to the grid lines is not limited to 45° or 135°, but will be more diverse. The number of edges in the graph will also increase, which can improve the path results obtained by the DBA algorithm. However, this will also come with an increase in computation time. The experiments and analysis will be discussed in Section 4.

To address the inclusion of suitable diagonals, for simplicity, we approximate the 3D representation by a 2D one. This allows all of the grid cells to be chosen to be congruent in the sense that the directions and lengths of their corresponding sides and diagonals are the same. Note that this 2D assumption is not a good approximation in areas close to the poles. However, most cables in the world are significantly far from the poles, so this approximation is generally useful [10]. For regions of interest to us, the latitude variation is not enough to cause significant variation in the lengths of the sides of the grid cells.

We consider different graphs with diagonals in different directions. It is generally expected that, because of the restricted directions available in the graph, for path planning based on DBA, different gridded graphs will result in quite different results. Evidently, as demonstrated in Section 1, for DBA, in general, cable direction is a factor in choosing effective gridded graphs.

For a fair comparison, when we compare FMM against DBA, the sensitivity to the cable direction needs to be considered as it may significantly affect the DBA performance. In this paper, for the use of DBA, we only allow the addition of edges that are diagonals of the grid cells in addition to the sides of the grid cells. For DBA, adding edges can improve the resulting path planning quality. However, it also leads to increased runtime as the number of edges |*E*| increases. For that reason, we will simultaneously present the results for runtime and cable path planning quality. The quality of the cable path is the primary criterion for evaluating the effectiveness of algorithms. By incorporating the factor of runtime into our comparisons, we aim to provide the industry with a reference point. When dealing with large datasets, they need to consider the cost implications associated with runtime. A detailed discussion is provided again in Section 5.

## 4 Numerical results

**Fig 3:**
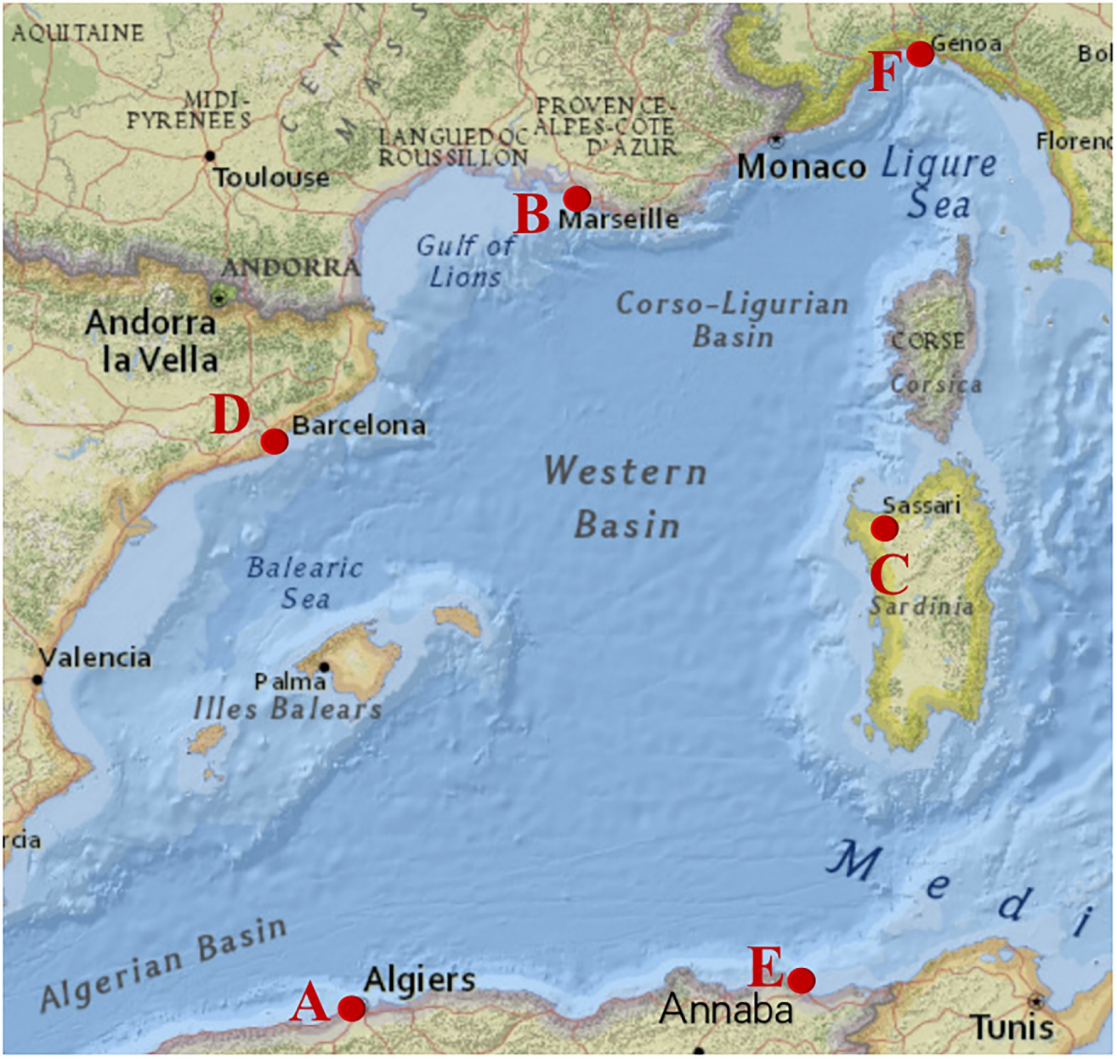
Target region D. Source: USGS National Map Viewer.

In this section, we present realistic numerical results for comparisons between FMM, DBA, and the great circle-based approach. Our examples are all conducted in the region D described in terms of the northwest corner at 45.000°N, 0.000°E and the southeast corner at 36.000°N, 11.000°E. The bathymetric data is taken from the Global Multi-Resolution Topography synthesis [24]. There are five terminal nodes in this area, as shown in Fig 3, Algiers (36.761°N, 3.074°E), Marseille (43.297°N, 5.359°E), Sardegna (40.557°N, 8.312°E), Barcelona (41.368°N, 2.190°E), and Annaba (36.928°N, 7.760°E), denoted by A, B, C, D, and E, respectively. Based on the investigation of the total length and cost of three undersea cable systems (i.e., Medloop, Med Cable Network, Bluemed) in the Mediterranean area, we set the average basic cost of the cable as $25,000 per km. In addition to the basic construction cost, we incorporate a weighted cost factor that takes account of sea depth and seabed slope during the path planning process. For detailed information on these weighted factors, readers are referred to [1, 6]. This study aims to compare different path-planning methods using identical input data. Performance comparisons are based on cable cost and runtime outcomes.

All simulations in this section are in Matlab on a computer with a 4.20-GHz Intel Core i7-7700K CPU. Specifically, to find the optimal cable path efficiently for both FMM and DBA, we adopt a mixed programming approach of Matlab with packages for the core computational codes of FMM and DBA written in C/C++. We use a *mexFunction* to link the Matlab scripting language and the compiled C/C++ language.

### 4.1 Runtime comparison for FMM and DBA

**Fig 4:**
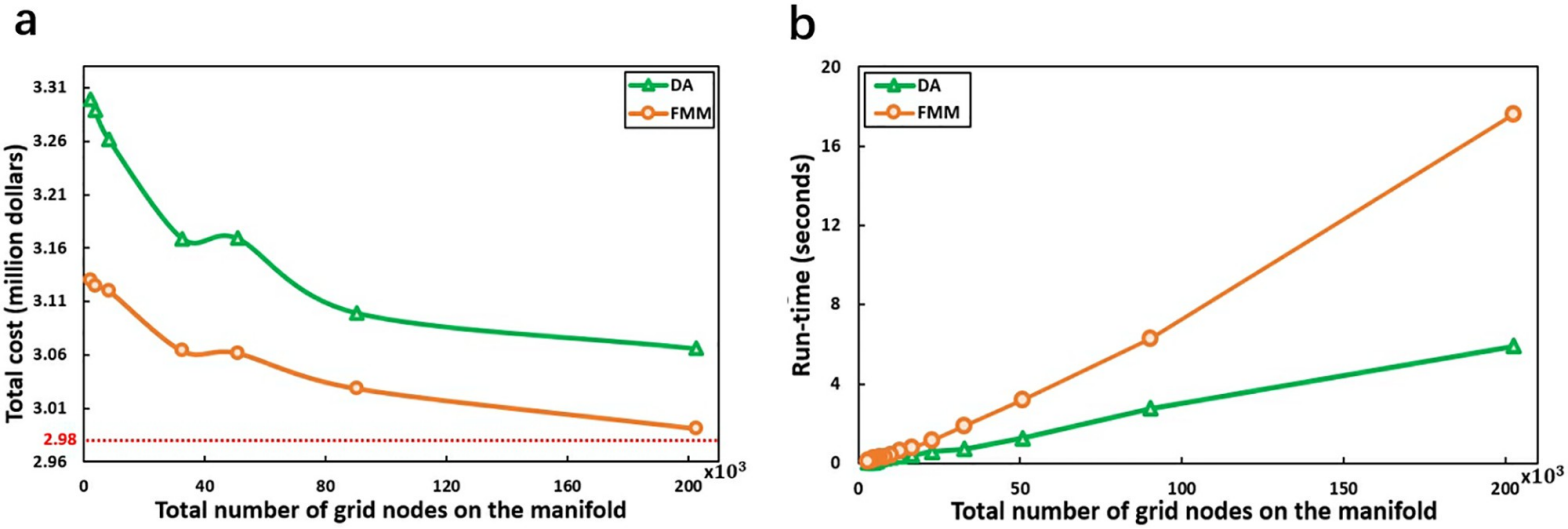
Comparisons between FMM and DBA. (a) The results of FMM and DBA based on different resolutions. (b) The runtime of FMM and DBA.

We start by running FMM at the highest resolution in which the average distance between two adjacent grid vertices is about 0.925km. As we expect it to give the best solution, we consider the result from the highest resolution to be the benchmark. To compare the runtime for FMM and DBA, we apply the two algorithms in different resolution maps. The low resolution is produced by sampling the highest resolution map at different sample spacings. As the resolution increases, the number of grid nodes (|*N*|) increases. Table 2 provides the runtime of FMM and DBA at different resolution maps. We observe that, as expected, the quality of the cable path results of the two approaches improves with |*N*|, as shown in Fig 4a. The runtime of FMM grows logarithmically with |*N*|, which is consistent with the discussion in Section 1. As we have discussed previously, the runtime complexity of DBA is Θ(|*E*| + |*N*| log |*N*|) (Fredman and Tarjan 1984). In our examples, the edges are only between adjacent nodes so the graph is incomplete. The size of |*E*| also depends on |*N*|, as follows,

$$\begin{array}{c} |E|=L*(H-1)+(L-1)*H, \end{array}$$ (5)

where *L* and *H* respectively represent the number of nodes per row and per column in the graph, and |*N*| = *L* * *H*. However, |*E*| is much smaller than |*N*| log |*N*|, when the number of grid nodes |*N*| is in the range 2.1 * 10^3^ to 2.1 * 10^5^. Therefore, the dominant term in the runtime calculation for DBA is |*N*| log |*N*|. Although the runtime complexity of FMM is similar to DBA’s, the relevant coefficients are different, as shown in Fig 4b where we see that the runtime of FMM grows faster than that of DBA.

**Table 2:** FMM versus DBA.

Resolution (km)	Number of grid nodes	Method	Total cost (million $)	Runtime (s)
18.5	2.10 * 10^3^	DBA	3.098	0.150
		FMM	3.03	0.25
13.89	3.70 * 10^3^	DBA	3.008	0.301
		FMM	3.024	0.47
9.25	1.00 * 10^4^	DBA	3.251	0.613
		FMM	3.12	0.953
4.62	3.26 * 10^4^	DBA	3.158	0.901
		FMM	3.064	2.064
3.70	5.08 * 10^4^	DBA	3.118	1.271
		FMM	3.062	3.203
2.77	9.02 * 10^4^	DBA	3.079	2.453
		FMM	3.028	6.781
1.85	2.03 * 10^5^	DBA	3.056	6.902
		FMM	2.991	20.476

*The total cost includes risk penalties.

The results indicate that, for realistic point-to-point path planning problems, DBA has a lower runtime than FMM, consistent with our previous discussion in Section 2. In the examples presented in this paper, due to limitations in publicly available ocean data, the resolution of the graph we used was not high. As a result, the difference in runtime taken by FMM and DBA was not significant, which is in the order of seconds.

### 4.2 Sensitivity to diagonal configurations

**Fig 5:**
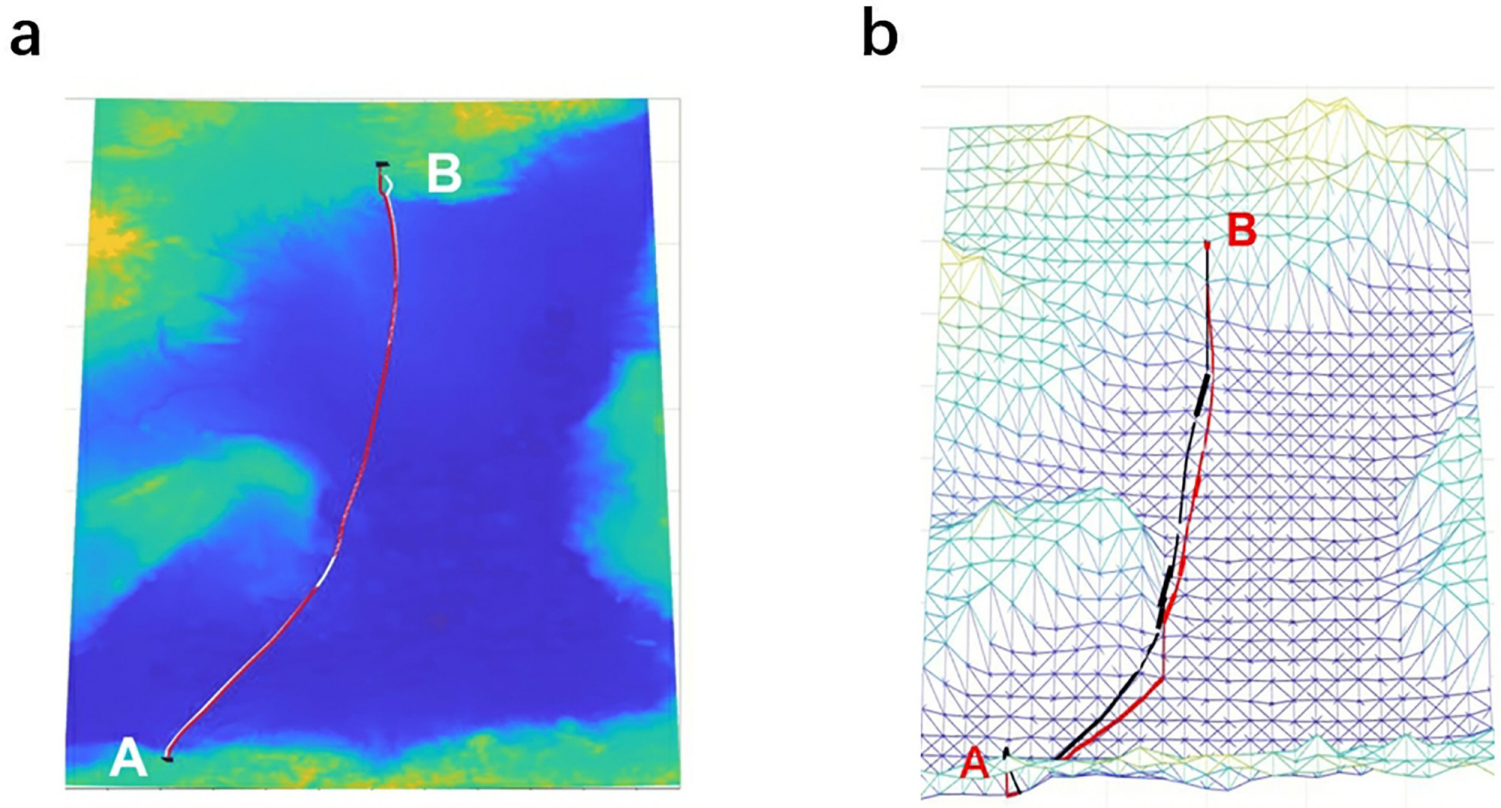
Cable path results of FMM in the two triangulated manifolds TM-I and TM-II. (a) The average distance between two adjacent grid vertices is 0.925km (red line for TM-I, white line for TM-II). (b) The average distance between two adjacent grid vertices is 27km (red line for TM-I, black line for TM-II).

**Fig 6:**
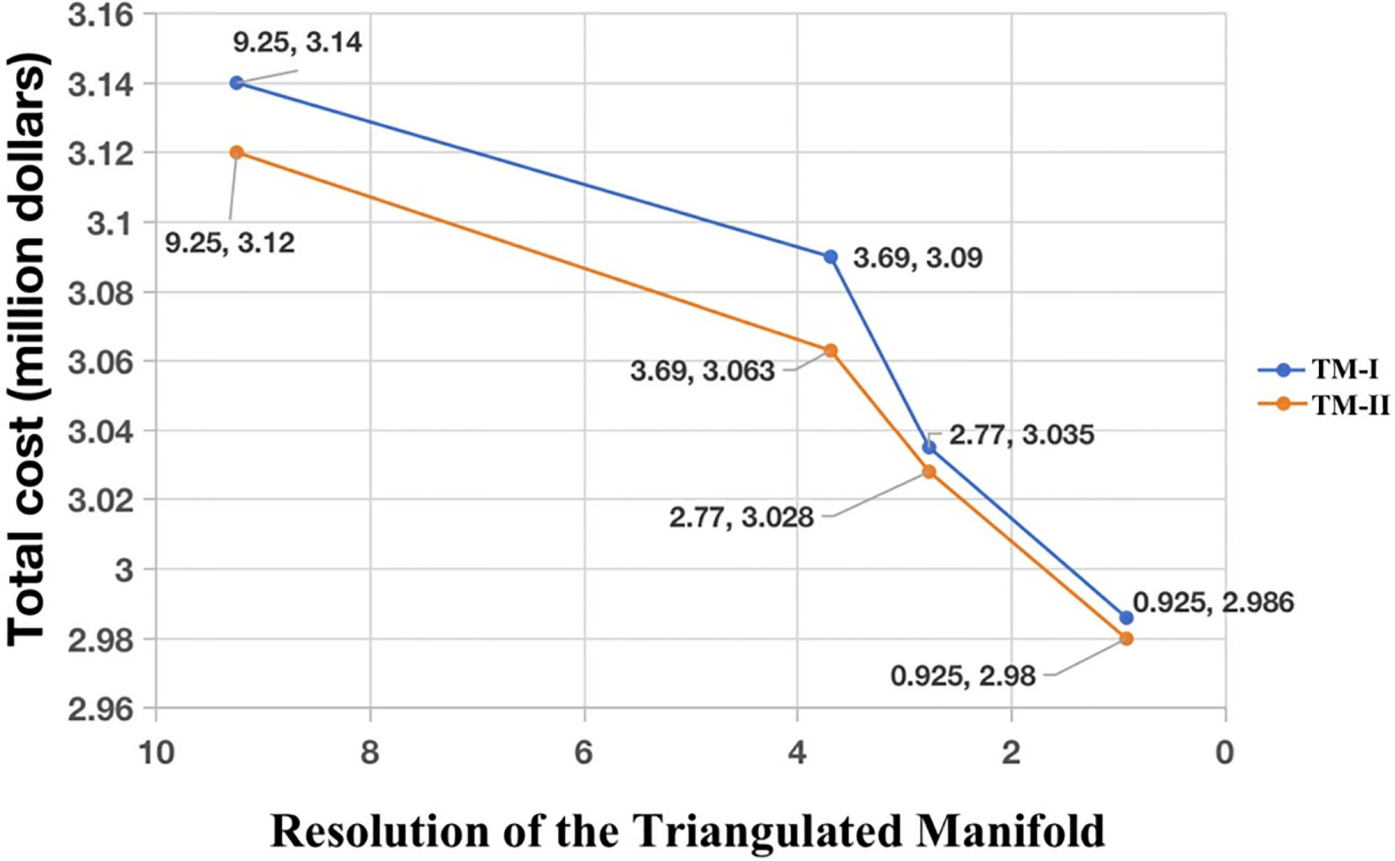
Weighted cost results of FMM in the two triangulated manifolds TM-I and TM-II as functions of resolution.

For FMM, in addition to varying resolutions, different ways of triangulating the manifold may affect the cable path planning result. Nevertheless, because FMM solves the Eikonal equation, which does not rely on the gridding, effects due to gridding should be just numerical artifacts rather than systematic errors related to the grid. We consider two different triangulated manifolds shown in Fig 2a and 2b. For the highest resolution manifold, the results from the two manifolds overlap almost completely, as shown in Fig 5a. The total cost of the optimal cable path that connects nodes A and B obtained by FMM in the two manifolds are $2.980 million and $2.986 million. Although, as the resolution decreases, the difference between the two paths obtained from the two manifolds gradually becomes more apparent, it is still not very significant, as shown in Fig 5b. This is consistent with the above-mentioned fact that FMM does not depend on the gridding. The cost results (based on cable length risk penalties) of the various cable paths obtained by FMM on different resolution maps are shown in Table 3 and Fig 6.

**Table 3:** Results of FMM in the two triangulated manifolds TM-I and TM-II.

Resolution (km)	Triangulation	Total cost (million $)	Difference (million $)
9.25	I	3.14	0.02
	II	3.12	
3.69	I	3.09	0.068
	II	3.063	
2.77	I	3.035	0.007
	II	3.028	
0.925	I	2.986	0.006
	II	2.98	

**Fig 7:**
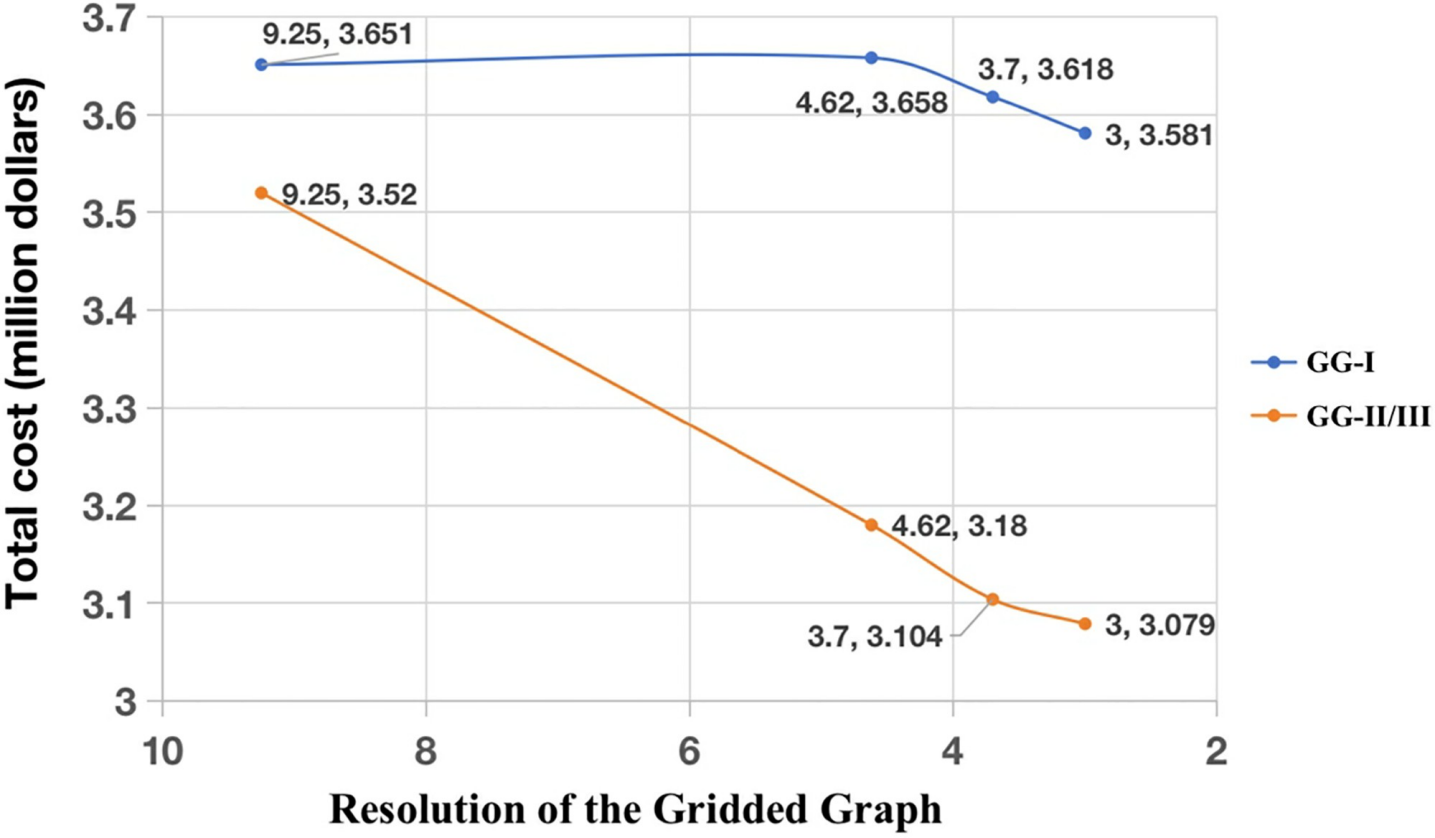
Weighted cost results of DBA in different gridded graphs GG-I and GG-II/III as functions of resolution.

**Fig 8:**
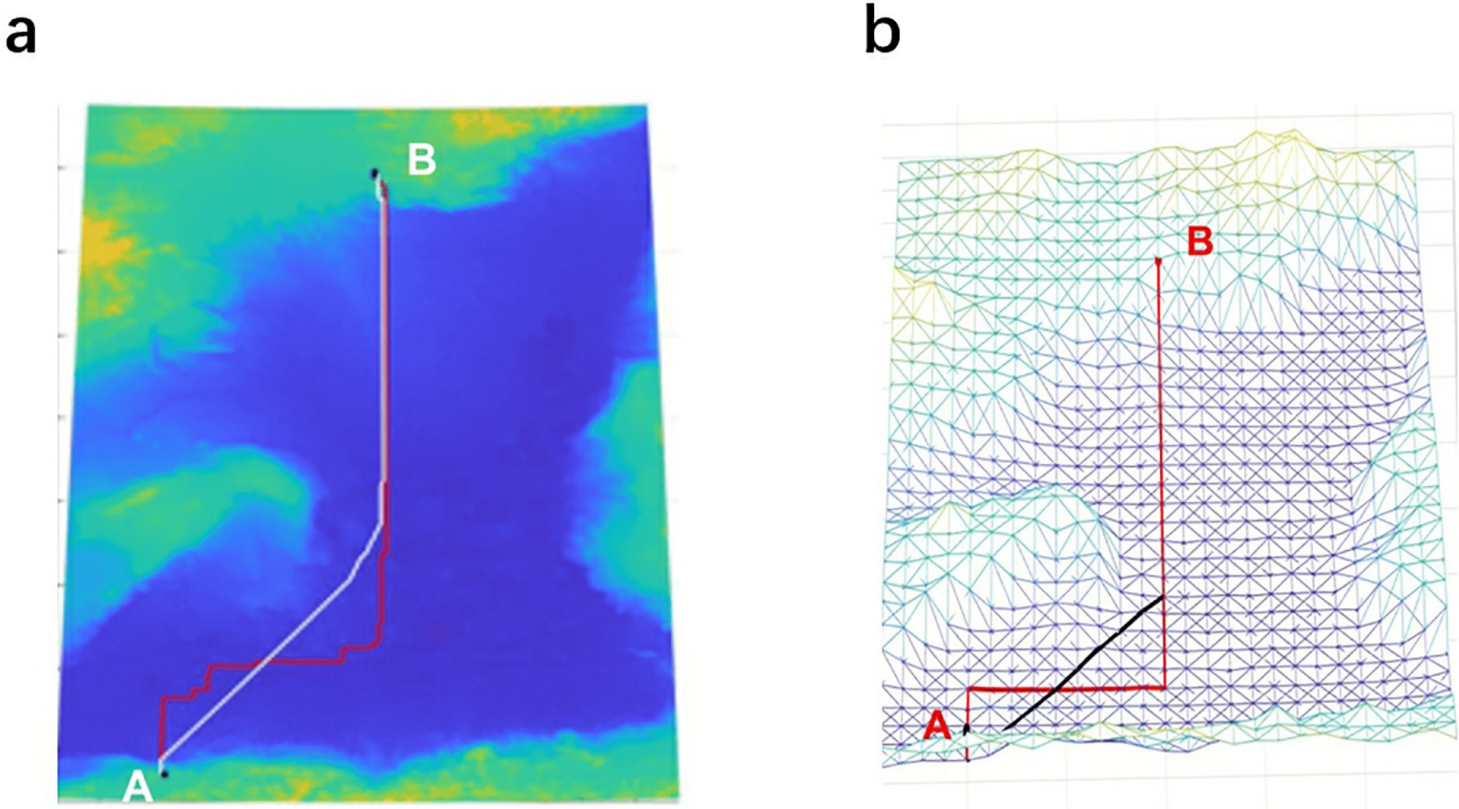
Cable path results of DBA in the two gridded graphs GG-I and GG-II. (a) The average distance between two adjacent grid vertices is 3km (red line for GG-I, white line for GG-II, and III). (b) The average distance between two adjacent grid vertices is 27km (red line for GG-I, black line for GG-II, and III).

For DBA, the results obtained based on GG-I and GG-II have obvious differences, regardless of the resolution, as shown in Table 4 and Fig 7. The cable path results based on GG-II are the same as those obtained in GG-III (Fig 2c), so we only present these results for the one path based on GG-II. These results are the same because the direction of the cable path generally for its entire length is either southwest to the northeast or due north, so the southeast to northwest diagonals of GG-I are never used when the graph is based on two diagonals per grid cell. The cable path results obtained in GG-I and GG-II with two different resolutions are presented in Fig 8. These results illustrate the fact that DBA is sensitive to triangulation. In addition to adding diagonals within a single grid cell, we also incorporate diagonals across two adjacent grid cells. This diversifies the angles of the diagonals and increases their quantity. This approach improves DBA results by $0.075 million compared to GG-II/III, albeit with a runtime increase of 0.022s. However, adding diagonals across adjacent grids may introduce some information loss in a given resolution map. Additionally, if the cable path direction does not correspond to the direction of the newly added diagonals, the quality of the cable path may not improve. Therefore, the decision to add additional diagonals should consider the direction of the cable path.

**Table 4:** Results of DBA in the different gridded graphs GG-I and GG-II/III.

Resolution (km)	Gridded graph	Total cost (million $)	Difference (million $)
9.25	I	3.651	0.401
	II/III	3.25	
4.62	I	3.658	0.478
	II/III	3.18	
3.70	I	3.618	0.514
	II/III	3.104	
3	I	3.581	0.502
	II/III	3.079	

### 4.3 Sensitivity to cable direction

In addition to the direction and number of the diagonals or the triangulation ways, cable direction also influences the performance of FMM and DBA for cable direction. To assess this, we perform cable path planning for three pairs of endpoints by applying FMM and DBA. We also provide path planning results based on the great circle-based method for reference.

**Fig 9:**
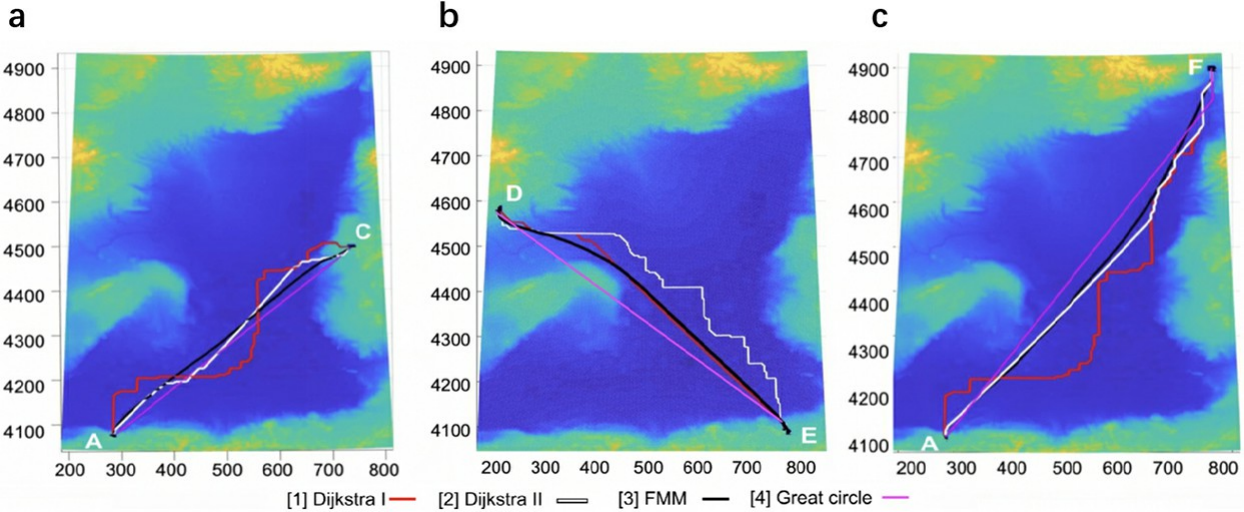
Comparisons of FMM, DBA, and the great circle-based method. (a) The first pair of endpoints (A, C). (b) The second pair of endpoints (D, E). (c) The third pair of endpoints (A, F).

The overall cable direction between the first pair of endpoints (A, C) is from southwest, node A, to northeast, node C, as shown in Fig 9a. The cable direction is more consistent with the direction of diagonals in GG-II & III than in GG-I. The results for the first pair of endpoints using FMM and DBA are shown in Fig 9a and Table 5. For DBA, the path obtained based on GG-II& III has a lower optimal cost value by 23.58% than that of the path based on GG-I.

**Table 5:** Comparison of FMM, DBA and the great circle-based method for the first pair of endpoints.

Scenes	Methods	Total cost (million $)	Deviation from benchmark	Path	Time(s)
GG-I	DBA	3.383	28.09%	Fig 9a-[1]	0.051
GG-II & GG-III	DBA	2.760	4.51%	Fig 9a-[2]	0.053
TM-I&II	FMM	2.640	benchmark	Fig 9a-[3]	2.248
\	Great circle	2.984	12.99%	Fig 9a-[4]	\

The cable direction between the second pair of endpoints (D, E) is from southeast, node E, to northwest, node D, as shown in Fig 9b. The cable direction is more consistent with the direction of diagonals in GG-I & III than in GG-II. The results for the second pair of endpoints obtained by FMM and DBA are shown in Fig 9b and Table 6. For DBA, the path obtained based on GG-I has a lower optimal cost value by 19.33% than that of the path based on GG-II. In this case, the results obtained based on the Great-circle method are not adopted due to a portion of the path planning extending onto land.

**Table 6:** Comparison of FMM, DBA, and the great circle-based method for the second pair of endpoints.

Scenes	Methods	Total cost (million $)	Deviation from benchmark	Path	Time(s)
GG-I & GG-III	DBA	2.860	2.41%	Fig 9b-[1]	0.061
GG-II	DBA	3.569	21.74%	Fig 9b-[2]	0.055
TM-I&II	FMM	2.790	benchmark	Fig 9b-[3]	2.054
\	Great circle	cannot be used	\	Fig 9b-[4]	\

The direction of the cable between the third pair of endpoints (A, F) is from the southwest node A to the northeast node F, as depicted in Fig 9c. However, the direction of the third cable is closer to the north than the direction of the first pair of nodes. Fig 9c and Table 7 present the results obtained by FMM and DBA for the third pair of endpoints. The path obtained using DBA in GG-II has a lower optimal cost value of 28.35% compared to the path obtained in GG-I & III. However, the optimal cost of the cable path obtained in GG-II is 3.05% worse than the benchmark. The performance of DBA in the third case is inferior to that in the first case, as the cable’s orientation aligns more precisely with the diagonal direction in the first scenario.

**Table 7:** Comparison of FMM, DBA, and the great circle-based method for the third pair of endpoints.

Scenes	Methods	Total cost (million $)	Deviation from benchmark	Path	Time(s)
GG-I	DBA	3.745	31.4%	Fig 9c-[1]	0.064
GG-II & GG-III	DBA	2.935	3.05%	Fig 9c-[2]	0.059
TM-I&II	FMM	2.850	benchmark	Fig 9c-[3]	2.161
\	Great circle	3.005	6.92%	Fig 9c-[4]	\

Path planning based on the great circle-based method does not consider the complexity of the seabed surface, human activities, and natural disasters. Its resultant path is a smooth arc that is the shortest distance on a sphere (representing the Earth’s surface) between start-end terminals and is not intended to bypass high-risk or high-cost areas.

In the example shown in Fig 11, we assume that there is a fishing area that needs to be bypassed by the planned cable. In this case, the best performance of DBA is expected to occur in the case of two diagonals in each grid cell because an additional option is available, allowing for significant direction changes in the cable path.

**Fig 10:**
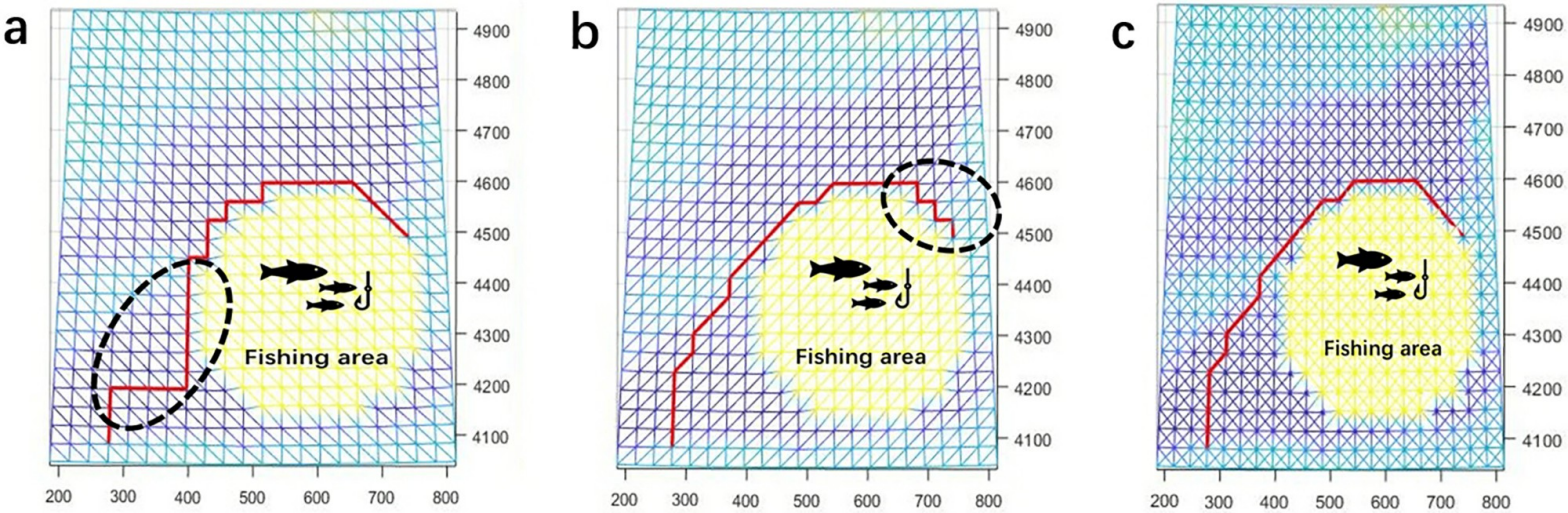


To illustrate this, we run DBA in the low-resolution case to show the difference in the results based on the three cases. As shown in Fig 10, in GG-I (Fig 10a) and GG-II (Fig 10b), the cable path in the areas represented by dotted circles is not as good as a result from GG-III (Fig 10c) because both diagonals are required.

**Fig 11:**
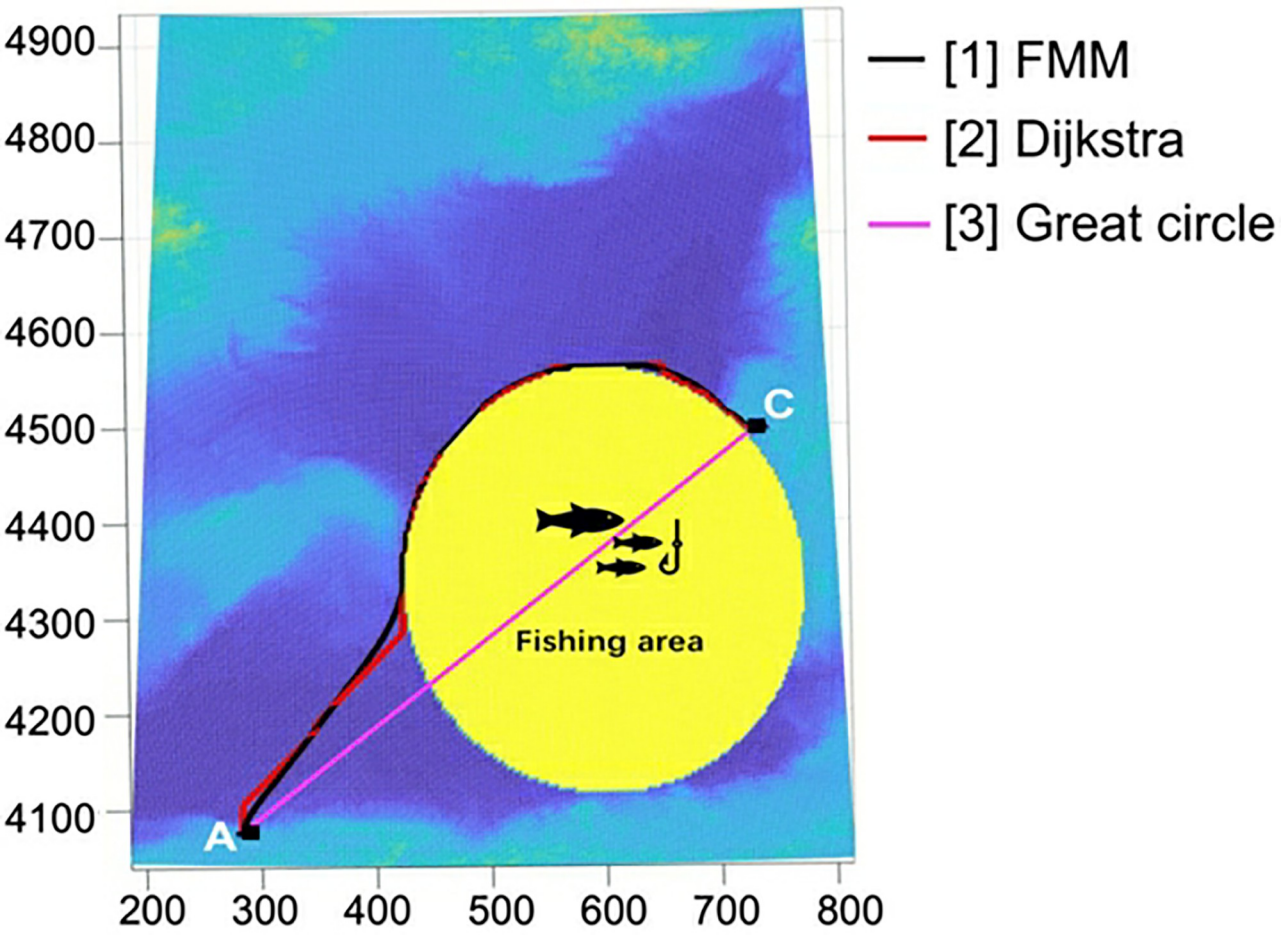
Comparisons of FMM, DBA, and the great circle-based method (with an obstacle).

Then, we apply DBA to GG-III with the highest resolution and compare their results against the FMM benchmark. The results are shown in Table 8 and Fig 11. As expected, the best result of DBA is obtained from GG-III, which is only 1.07% in cost worse than the benchmark, while the results obtained in GG-I and GG-II are 13.83% and 9.87% worse than the benchmark, respectively. In this case, the results obtained based on the Great-circle method are not adopted due to a portion of the path planning extending onto the obstacle area.

**Table 8:** Comparison of FMM, DBA, and the great circle-based method (with an obstacle).

Scenes	Methods	Total cost (million $)	Optimal deviation	Path	Time(s)
GG-I	DBA	3.711	13.83%	\	0.078
GG-II	DBA	3.582	9.87%	\	0.078
GG-III	DBA	3.295	1.07%	Fig 11-[2]	0.187
TM-I & TM-II	FMM	3.260	benchmark	Fig 11-[1]	2.359
\	Great circle	cannot be used	\	Fig 11-[3]	\

## 5 Discussion

In all the experiments we conducted, FMM consistently achieved lower path costs than DBA, with the weighted cost of cable from FMM being 2.1% to 28% less than that of DBA. Although DBA is faster than FMM at the same resolution, including scenarios with two diagonals in each grid cell, the difference is not significant due to limitations in publicly available ocean data, as discussed in Section 4. As we demonstrate in this paper, FMM has an advantage over DBA in waters where only low-resolution data is available, and this comprises most of the ocean. Based on available data, our experimental results indicate that the runtime for FMM is 1.5 to 3 times longer than that of DBA. The results of DBA across different graphs with various diagonal configurations and cable direction scenarios show that DBA is more sensitive to diagonal configurations and cable directions, whereas FMM is less sensitive and provides lower-cost cable paths. Our experiments are based only on publicly available data and for our purpose of algorithm comparison, we use a kilometer-scale resolution which, as mentioned above, is the available deep waters resolution. As 90% of the entire ocean area is in deep waters, it seems reasonable for our purposes of algorithm comparison using a kilometer-scale resolution.

One limitation of automatic algorithms is their reliance on publicly accessible data, which may restrict the quality of their cable path outcomes. However, by incorporating additional privately collected data from areas near the path generated by automatic algorithms and using the algorithms again based on the additional data, it is possible to enhance the path quality to some extent. It is important to note, however, that it is difficult to obtain very high-resolution data in deep waters. In the examples presented in this paper, due to limitations in publicly available ocean data, the resolution of the graph we used was not high. As a result, the difference in runtime taken by FMM and DBA was not significant, which is in the order of seconds. In such cases, the FMM algorithm exhibits clear advantages because, in practice, cable path planning projects normally take weeks or months, and such short running times make no difference.

However, when dealing with design problems of cable systems over large areas that involve many cables, branching units, and cable landing stations, where data volume becomes substantial and the graph that represents the data has billions of nodes, the running time may become an issue that needs to be considered to address the scalability limitation of FMM. This may become a challenge if the target region is very large and the data resolution is also very high. However, the resolution of publicly available data is not very high, so the scalability limitation will not inhibit generation of the first path. Then, when more data of higher resolution is collected, it is collected only in the relevant region near the initial path, so the target region will not be prohibitively large.

For applications where the resolution is very high so that the runtime is prohibitive, the use of DBA may be an option. However, FMM can still be used with a multi-resolution approach that saves runtime by increasing the resolution of the set of data at or near a path obtained by lower-resolution data using FMM [25]. In addition, parallel computing of FMM in [26] can also be used to reduce runtime if necessary. Recall that we mentioned in Section 2, that also DBA with parallel processing was used by [16] for cable path planning.

Another limitation of the automatic cable path planning algorithms is that they have not considered the bending fitness of a cable. Cables are designed and manufactured with specific bending capabilities, which are typically provided by the cable manufacturer [14]. These specifications outline the acceptable limits for bending radius. During the cable installation process, a curvature is formed due to the need to navigate around obstacles. The radius of this curvature must not exceed the bending radius limitation of the cable [14, 27]. The curvature in the cable path is a critical factor that must be considered to ensure the cable’s integrity and functionality. For this reason, the existing academic published work on cable path planning would require post-process manual smoothing.

We would like to clarify that the algorithms and solutions for automated path-planning are not aimed at completely replacing the manual approach used currently by surveyors and path-planning experts. Rather, such algorithms and solutions provide an initially optimized path to guide designers to areas of the path where further data needs to be collected and/or to improve the path manually. Note that the paths provided automatically (especially using DBA) may not satisfy the cable bending stiffness that is infeasible for laying cables, as mentioned above, but these can be corrected manually. Then, where more data is available, automated path planning algorithms can be used by the industry as a benchmark to complement their manual approach and achieve significant cost savings for all stakeholders. Automated path-planning algorithms will benefit from the inclusion of new considerations learned from human experts and vice versa. Experts will improve their design with the help of the automatic path planning results. Combining automated path-planning algorithms with manual path-planning is beneficial for the industry. In this context, when dealing with low-resolution areas, manual adjustments have the potential to rectify imperfections in cable paths generated by automated algorithms. These imperfections may include bending limitations and the omission of risk areas.

Overall, the use of automatic approaches can enhance and reduce costs in the commonly used manual approach by cable system planners, designers, and surveyors and improve submarine cable path planning by optimizing objectives associated with cost and risk, as well as meeting the QoS requirements. With the use of such software, both the cost and time for the cable path planning process can be reduced. Another important advantage of the automated path planning method we propose is that it allows fine-tuning, under the control of the human user, of the balance between risk and cost by using Pareto optimality.

## 6 Conclusion

In this paper, we considered an application of Dijkstra’s algorithm (designated DBA) for cable path planning by allowing the path not to be restricted to grid edges. We have conducted a thorough and fair comparison of DBA, FMM, and a great circle-based method, evaluating their performance across various scenarios. Considerations affecting the performance of DBA and FMM path planning algorithms, such as map resolution, diagonal configurations, and cable direction, have also been studied. The performance of these automated methods has been evaluated in terms of the path quality measured by the optimal value of the weighted cost function and runtime. From the experiments with various diagonal configurations and triangulation methods, we have observed that DBA is sensitive to the choice of diagonal configuration in the graph and cable direction, while FMM is not. The results show that FMM provides the optimal cable path with the lowest weighted cost among the three methods. Specifically, based on available data, the weighted cost of the cable from FMM is 2.1% to 28% lower than that of DBA, although FMM’s runtime is 1.5 to 3 times longer than DBA’s. Overall, this paper provides the industry with useful reference for choosing automated path-planning algorithms. Additionally, the limitations and weaknesses of both FMM and DBA have been discussed. Despite the constraints of automated path-planning methods, they can still assist surveyors and designers by offering an initial path, enabling them to focus their data collection on areas near the chosen path.

In scenarios involving path planning across extensive areas with substantial data, particularly for ultra-long (over 10,000 km) and high-resolution cable paths, FMM may require an excessive runtime and storage capacity. In this context, parallel computing and multi-resolution analysis emerge as promising techniques to address this computationally intensive challenge [15, 28–30]. In addition, we may consider using a combination of FMM and DBA where each is used in a parallel algorithm in different regions to save running time without adversely affecting the result achieved in terms of the weighted cost of the optimal path.
